# Intact spatial learning in Fmr1^–/*y*^, Syngap1^+/–^, and Grin2b^+/–^ rat models of neurodevelopmental disorders

**DOI:** 10.3389/fnbeh.2026.1929983

**Published:** 2026-09-25

**Authors:** Anuj Vadher, Adrian J. Duszkiewicz, Emma R. Wood, Paul A. Dudchenko

**Affiliations:** 1 School of Psychology, University of Stirling, Stirling, United Kingdom; 2 Division of Neuroscience, University of Manchester, Manchester, United Kingdom; 3 Institute of Neuroscience and Cardiovascular Medicine, University of Edinburgh, Edinburgh, United Kingdom

**Keywords:** autism spectrum disorder (ASD), intellectual disabilities, developmental disabilities, landmark, spatial memory, water maze (WM)

## Abstract

**Introduction:**

FMR1, SYNGAP1, and GRIN2B gene mutations Q8 are known causes of mild to severe intellectual disability (ID) in humans, with affected individuals frequently co-diagnosed with autism spectrum disorder (ASD). These three genes influence molecular mechanisms underlying synaptic plasticity, and thus spatial memory behaviors have been of interest in studying rodent models of these mutations. However, evidence of spatial memory impairment across these models is inconsistent, with some studies reporting deficits and others finding none.

**Method:**

In the present study, we used a variant of the Morris water maze that allowed explicit stimulus control of visual landmarks and examined the use of individual visual cues to navigate, using *Fmr1*^–/y^, *Syngap1*^+/–^, and *Grin2b^+/–^* rat models.

**Results:**

We found 1) strong landmark control of spatial behavior and 2) intact learning in all three rat models.

**Discussion:**

Of general significance, our results also suggest that rats use a spatial strategy that relies on individual landmarks (at least in a directional water maze), as opposed to the configuration of landmarks, to identify the goal location.

## 1 Introduction

Approximately 2–3% of the general population are affected by intellectual disability (ID), often co-diagnosed with autism spectrum disorder (ASD) or epilepsy (Jansen et al., 2023). Multiple genes associated with these conditions have been identified, and a promising way of assessing both the behavioral and neural phenotypes of these neurodevelopmental conditions is with rodent models. In this study, we focus on three such models: *Fmr1*^–/*y*^, *Syngap1*^+/–^, and *Grin2b^+/–^* rats. These are models of Fragile X syndrome, SYNGAP1-related ID, and GRIN2B-related neurodevelopmental disorder respectively. Fragile X Syndrome - in which a mutation of the Fragile X Messenger Ribonucleoprotein 1 (*FMR1*) gene on the X chromosome results in a CGG repeat expansion, leading to the loss of FMRP protein - is an inherited monogenic condition affecting 1 in 4000 males and 1 in 7000 females (Richter and Zhao, 2021). FMRP is strongly expressed in neurons within the cortex, hippocampus, thalamus, and cerebellum (Zorio et al., 2017), and alterations in spine formation and pruning have been described in both animal models and in human Fragile X syndrome (Irwin et al., 2000; Till et al., 2015; but see Nimchinsky et al., 2001), as well as abnormal synaptic plasticity and elevated protein synthesis in the former (Till et al., 2015). The Synaptic Ras GTPase-activating protein 1 (*SYNGAP1*) gene encodes for protein SynGAP1, found in the pre-synaptic density (Sabo et al., 2023). Mutations of this gene account for up to 1% of all non-syndromic intellectual disability. Finally, *GRIN2B* mutations can cause over or under expression of the GluN2B NMDA receptor subunit, and both *SYNGAP1* and the rarer *GRIN2B* disorder arise from *de novo* mutations (Gamache et al., 2020; Platzer and Lemke, 2018).

Despite impairments in intellectual ability present in these three conditions in humans - all are associated (at least in males) with IQs of 70 or below - the evidence of learning or memory impairments in rodent models has been mixed. The majority of studies thus far have been done in *Fmr1*^–/*y*^ mice and rats. For *Fmr1*^–/*y*^ mice, either modest or no impairments were observed in spatial learning compared with WT animals, as assessed in the water maze or radial arm maze (The Dutch-Belgian Fragile X Consortium, 1994; Willems et al., 1995; Peier et al., 2000; Kooy et al., 1996; D’Hooge et al., 1997; Paradee et al., 1999; Dobkin et al., 2000; Van Dam et al., 2000; Yan et al., 2004; Mineur et al., 2002; Baker et al., 2010).

Mixed results were also observed in *Fmr1*^–/*y*^ rats. For example, Tian et al. (2017) found *Fmr1*^–/*y*^ rats had fewer platform crossings in a water maze probe session compared to WT rats, but did not find any difference for quadrant occupancy. However, Till et al. (2015) failed to find any difference between the *Fmr1*^–/*y*^ and WT rats in the standard reference memory, reversal, or delayed matching to place tasks in the water maze. More recently, Ouardouz et al. (2025) found that FMR1 KO rats were comparable to WT rats in their shorter latencies to find an escape hole on a Barnes maze with training, though a subset of FMR1 KO rats employed a potentially non-spatial strategy.

Fewer previous studies have assessed spatial memory in rodent models of SYNGAP1 haploinsufficiency. Nakajima et al. (2019) found that *Syngap1*^+/–^ mice showed clear impairments on a T-maze alternation task relative to WT animals and also showed impaired spatial learning on a Barnes maze. Komiyama et al. (2002) and Singh et al. (2025) found that *Syngap1*^+/–^ mice tested in a water maze showed significantly less preference for the correct platform location on transfer (platform removal probe) than WT mice. Similarly, Muhia et al. (2010) report lower preference for the target quadrant in the *Syngap1*^+/–^ mice for the first 15 s of a probe trial, but over the full 60 s both the WT and the mutant mice show a similar level of preference. They also report impairment in *Syngap1*^+/–^ mice for the radial arm maze, with the mutant mice not improving upon training for the reference memory task, and making more errors for the working memory task (see also Guo et al., 2009). In *Syngap1*^+/–^ rats, Katsanevaki et al. (2024) found slightly higher latencies to find a hidden platform in a water maze compared to WT rats during acquisition, but no differences in platform preference during probe trials and in reversal learning. The authors did not find any impairment in spatial memory for *Syngap1^+/^*^Δ^ ^–GAP^ rats compared to WTs in the same task. More recently, Katsanevaki et al. (2025) showed that *Syngap1*^+/–^ mice acquired a Y-maze visual discrimination task as well as WT mice, but showed significant impairments relative to WT animals when the visual stimuli were made less discriminable, indicative of an impairment in visual acuity.

*GRIN2B* rodent models are less well characterized, but the studies that do exist provide mixed evidence of impaired spatial learning following reduction in the expression of the GluN2B protein. For example, earlier work by Shin et al. (2020) found no impairment in Morris water maze acquisition, probe trial performance, or reversal learning in *Grin2b*^+/*C*456*Y*^ mice (which had a 30–50% reduction in GluN2B expression) compared to WT mice. However, a previous study using the Morris water maze in a GluN2B-trp373 variant rat model found that the mutant rats had fewer platform crossings compared to WT rats (Wang et al., 2022). Similarly, Harvey et al. (2026) found that mice with a 50% reduction in GluN2B protein expression showed impairments in long-term potentiation, long-term depression, Morris water maze acquisition/probe trial bias, and Barnes maze acquisition/probe trial performance.

Overall the evidence for impaired spatial learning in *Fmr1*^–/*y*^ is inconsistent, while somewhat more consistent evidence exists in *Grin2B* and *Syngap1* mouse models (although not in rat models).

In the current study we assessed *Fmr1*^–/*y*^ rats, in which males have no expression of FMRP, and *Syngap1*^+/–^, and *Grin2b^+/–^* rats, which are heterozygous knockout models with 50% expression of the SynGAP1 and GluN2B proteins. The aim of the current experiment was to compare these three models in a version of the water maze in which 1) the use of a sense of direction was key, and 2) the use of distal landmarks was controlled. The rationale underpinning this effort was two fold. First, in recent electrophysiology work, we have observed that head direction cells - neurons which putatively underpin the rodent’s sense of direction - show significant alterations in *Fmr1*^–/*y*^ rats compared to WT rats (Moore et al., 2025). Thus, a task that taps the rats’ use of a direction sense may be sensitive to *Fmr1*^–/*y*^ deficits in particular, and it is of interest to compare *Fmr1*^–/*y*^ rats with additional models of ASD/ID. Second, in most previous experiments employing a water maze, the use of distal visual landmarks for learning of the platform location was assumed, but not tested directly (e.g., Van Dam et al., 2000). In order to demonstrate a spatial association between the submerged platform location and visual landmarks in the testing room, we describe the development of a task variant in which these were shifted in tandem relative to the testing room for every session (see also Rodrigo et al., 1997). In this approach, the only informative extra-maze cues in the testing environment were the ones that we specified.

A secondary aim that arose during the conduct of the experiment was whether individual landmarks were sufficient to guide spatial behavior. Some, but not all of the small number of previous studies that attempted to specify the landmarks available in a water maze have suggested that multiple landmarks may be necessary for the rat to learn. Fenton et al. (1994a), for example, tested animals in the dark with two or four illuminated cues spaced relatively equivalently around the maze perimeter. Relevant to the current study, they found that, for the four-cue trained rats, any subset of two cues was sufficient to maintain performance, as measured in latency to find the submerged platform. A subsequent study by Rodrigo et al. (1997) found that three of any four extramaze visual landmarks on which rats had been trained were sufficient to support spatial memory, while Prados and Trobalon (1998) showed better performance in probe trials when more than one, trained landmark was available. In contrast to these results, Nakazawa et al. (2002) found that mice showed a similar preference for the platform quadrant in a probe session when only one of four trained landmarks was available.

Our results show that WT Long Evans rats readily use specified visual landmarks, and even subsets of trained landmarks, to guide their spatial behavior and that *Fmr1*^–/*y*^, *Syngap1*^+/–^, and *Grin2b^+/–^* rats show few differences in this spatial learning compared to their WT littermates.

## 2 Materials and methods

### 2.1 Animals

All the subjects were Long Evans rats bred by the University of Edinburgh by the Simons Initiative for the Developing Brain (SIDB). 18 rats were used for the SynGAP1 experiment (WT = 11 males, *Syngap1*^+/–^ = 7 males), 16 for the FMRP experiment (WT = 8 males, *Fmr1^–/*y*^* = 8 males), and 12 for the GluN2B experiment [WT = 6 (3 males, 3 females), *Grin2b^+/–^* = 6 (3 males, 3 females)]. All the rats were housed with littermates (2–5 per cage) in mixed genotype cages and kept on a 12h light/dark cycle with *ad libitum* access to food and water. At the start of the experiment, all rats were at least 13 weeks old (*Syngap1*^+/–^: 21–23 weeks old, *Fmr1*^–/*y*^: 20 weeks old, *Grin2b^+/–^*: 13–14 weeks old). A traditional version of the water maze was used to pilot our testing, before we moved to a directional water maze for the main studies reported here.

### 2.2 Water maze

A 200 cm diameter pool, filled with water up to 28 cm for visible platform training trials and 32 cm for the rest of the trials was used. The water temperature was maintained at 27 ± 2° C, and made slightly opaque by the addition of 250 ml of latex additive. An Atlantis platform of 12 cm diameter was used for the experiment (Spooner et al., 1994). The platform was placed such that it was 2 cm above water for visible platform training trials, and 2 cm under water for the rest of the training trials. During probe trials, the platform remained under water, held by magnets, for 60 s and then rose up to 2 cm beneath the water surface. All the rats remained on the platform for 30 s after finding it. The pool was surrounded by a black curtains with four different cues hanging affixed to the maze-surrounding curtains [lantern (15 * 35 cm), white board (78 * 59 cm), hi-vis jacket (70 * 58 cm), sombrero hat (30 cm radius)]. A square grid of LEDs was fixed above the pool in the ceiling to illuminate the pool with an overhead camera at its center.

*Traditional water maze (pilot)* Rats were initially given 8 trials of visible platform training (data not shown) before being moved on to the submerged platform training. During each training trial, the rats had 120 s to find the platform after which they were guided by the experimenter to the platform. During probe trials, the platform remained under water for 60 s and then rose up to 2 cm beneath the water surface. All the rats remained on the platform for 30 s after finding it. For probe trials, the platform was pushed under water and held in place by magnets. This version of the maze was used for *Fmr1*^–/*y*^ rats initially, though an annular search strategy was observed. To encourage use of the landmarks, a second version of the water maze task was developed as described below.

*Directional water maze* To increase the cost of the animal’s platform search, we added eight transparent acrylic triangular wall inserts into the pool (height 60 cm; long sides 60 cm; vertex angle 10°), which divided the pool into eight equal sections opening into a central circle of 80 cm diameter (See Figure 1). Thus, the rat could not simply swim in a circle a fixed distance from the pool walls to find the submerged platform, but needed to swim into and out of each “alleyway” created by the inserts (see also Martin et al., 2003; Penley et al., 2013).

**FIGURE 1:**
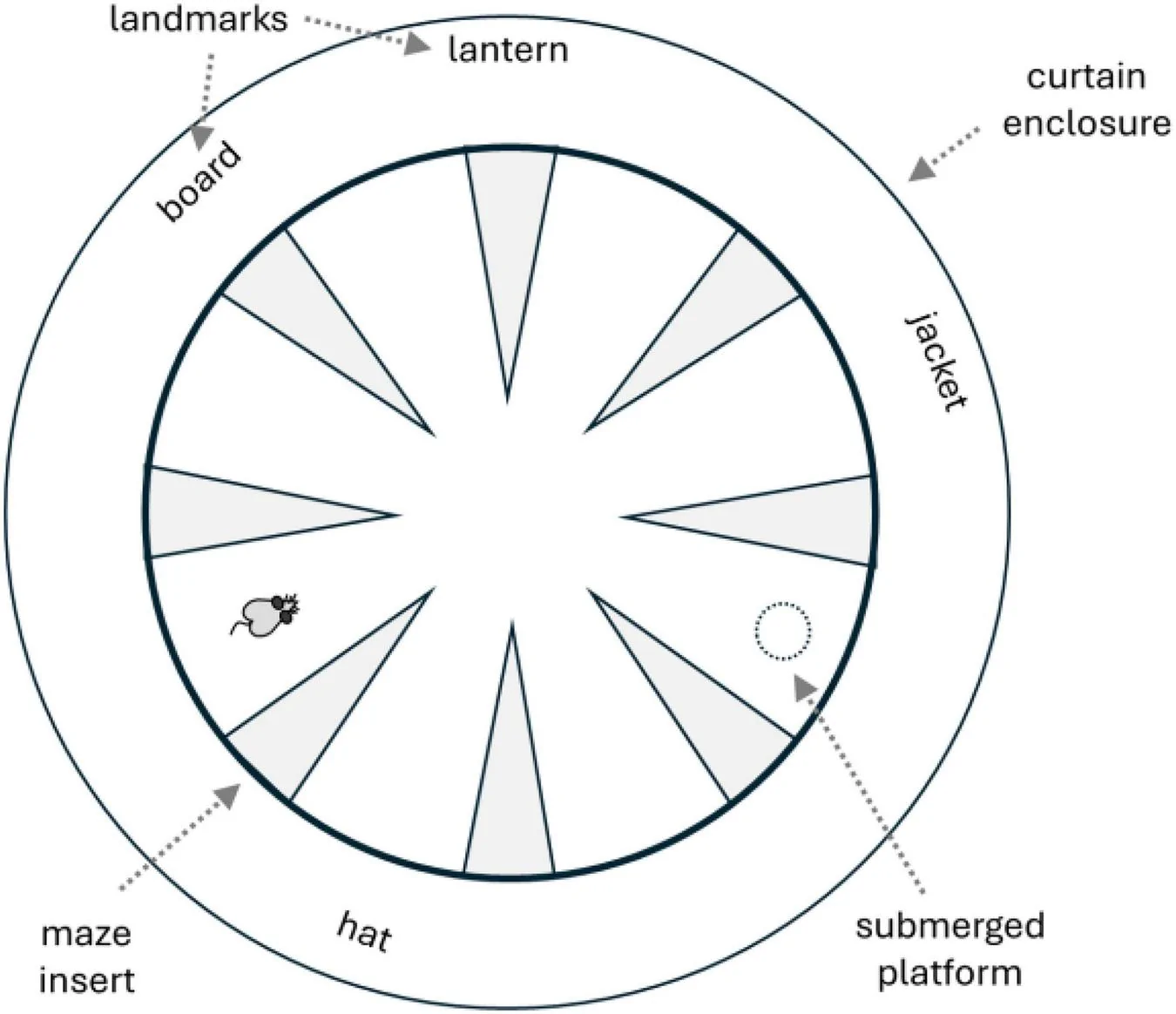
Schematic of the water maze. The water maze was equipped with inserts that restricted the rat to “alleyways,” one of which contained the submerged escape platform. The maze was curtained off from the remainder of the room with a black curtain, upon which were affixed four salient visual landmarks: a lantern, a white board, a hi-visibility jacket and a sombrero hat. The relative positions of these landmarks and the platform were fixed, but they were shifted (together) each day.

Two platform locations were used, with half the rats in a group experiencing the platform in one location and the other half experiencing the platform 180° from the other group. Each rat received four trials in a day, with different starting points for each trial (North, East, South, West). The order of starting points were pseudo-randomized for all the days. The rats were run in interleaved pairs with alternating trials between the two rats to provide a short gap (approximately 120 s) between successive swims for each rat. The experiments were conducted over 10 days, followed by additional days of probe trials where three out of the four landmarks were removed. At the beginning of each day, the landmarks and the platform were rotated together by a pseudorandom angle of an increment of 90°, but the landmarks and submerged platform always maintained their relative configuration. This was done for both training sessions and the probe sessions. Rats were given one session of testing each day, and thus the landmarks and platform were in a different location relative to any uncontrolled cues on adjacent days. Probe trials were conducted on the first trial of the day on days 2, 4, 6, 8, and 10. Days 1 and 2 were visible training trials, except for the 1st trial of day 2 which was a probe. From Day 3 onward, apart from the probe trials every alternate day, the rest of the trials were training trials. Following this training (days 1–10), rats were tested with probe trials with three out of the four landmarks removed. Different landmarks were removed for different groups of rats in order to test whether landmarks were used configurally or independently. Because it appeared as though some landmarks were better used by rats than others, for the *Fmr1*^–/*y*^ and *Grin2b^+/–^* rats, extra probe trials were added where different subsets of landmarks were removed to further understand this effect. These extra probes were preceded by 1 day of training trials with all landmarks present, and these trials were conducted at a variable interval from the end of day 11, ranging from 1 week to 4 weeks.

### 2.3 Analyses

All the trials were recorded with an overhead camera and Actimetrics water maze software (Lafayette, Indiana). The raw data with position tracking of the rats was analysed by a custom Python script. Since the water maze had eight alleyways, we calculated octant occupancy (in place of quadrant occupancy, which is standard practice) as a measure of memory. An octant was defined as the region within each of the alleyways created by the inserts. Occupancy was calculated as time spent within each octant as a proportion of time in all the octants. The first 60 s of all the probe trials were used for octant occupancy. To avoid inflating the occupancy of the octant at which the rat was placed in the pool, the time from the start of the trial until the rat first entered the central area (60 cm radius from the center of the pool) was excluded. A mixed-model ANOVA was conducted to analyse probe trial data of days 2–10. Mann-Whitney U tests were conducted to compare two groups during probe trials with a single landmark present. One sample *t*-tests with a chance level of 12.5% (that is, equal octant occupancy for all octants) were conducted to determine whether the rats performed above chance during a probe trial.

Data was analysed using Python (3.13.13) within a Conda environment; packages used include numpy (2.4.4), pandas (3.0.2), scipy (1.17.1), matplotlib (3.10.8), statsmodels (0.14.6), pingouin (0.6.1).

## 3 Results

### 3.1 Rats adopted an annular search strategy in an open water maze task

In a pilot experiment with a traditional water maze (that is, one without alleyways), both *Fmr1*^–/*y*^ and their WT rats developed a modest preference for the target quadrant (the quadrant which contained the hidden platform on all probe trials) across testing (Figure 2; *T*-test against chance performance: Day 14 - WT: *t*(4) = 2.47, *p* = 0.035, *Fmr1*^–/*y*^: *t*(4) = 1.74, *p* = 0.079; probe sessions were replaced with a training trial on days 6 and 7 in an attempt to improve performance). Rats developed an annular search strategy during probe trials, swimming in circles at approximately the correct distance from the wall. Radial band analysis confirmed significant (compared with chance) preference for the 60–80 cm band in both genotypes (Average occupancy of the last 5 probe trials compared to chance level - WT: *t*(4) = 2.81, *p* = 0.024, *Fmr1*^–/*y*^: *t*(4) = 2.99, *p* = 0.02). To combat this, for the main experiments of this paper inserts were added to the maze and all three genotypes were assessed with this maze configuration.

**FIGURE 2:**
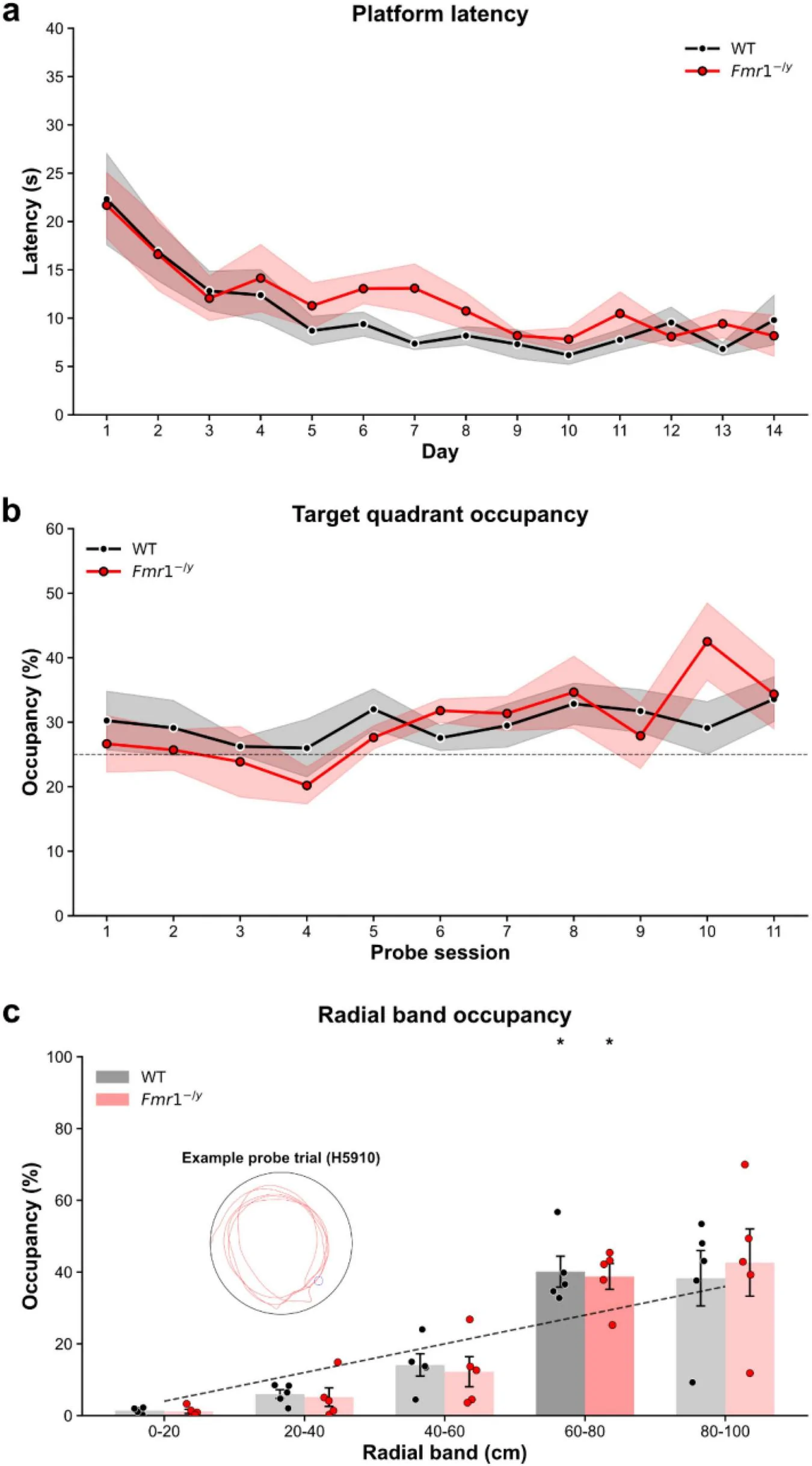
Performance of both *Fmr1*^–/y^ and WT rats in a traditional (open) water maze. **(a)** Latency to find the submerged platform decreased across training days. **(b)** Both *Fmr1*^–/y^ and WT rats developed a modest preference for the platform quadrant in daily probe sessions in which the platform was not available. **(c)** Both groups of animals appeared to develop an annular search strategy (see inset for example) whereby they spend the majority of their search time a fixed distance from the platform walls. Dashed line indicates chance-level occupancy.

### 3.2 *Fmr1*^–/*y*^ rats show robust learning in a directional water maze task

Across 10 days of training, both Fmr1^–/*y*^ and WT rats showed a decrease in time to find the hidden platform (main effect of training day: *F*(9,126) = 39.66, *p* < 0.001; see Figure 3a). There was a small but significant advantage for Fmr1^–/*y*^ rats compared to WT rats in task acquisition [*F*(1,14) = 5.87, *p* = 0.03], potentially influenced by slower latencies to find the platform for the WT rats on day 3 (interaction between genotype and days: [*F*(9,126) = 2.06, *p* = 0.038].

**FIGURE 3:**
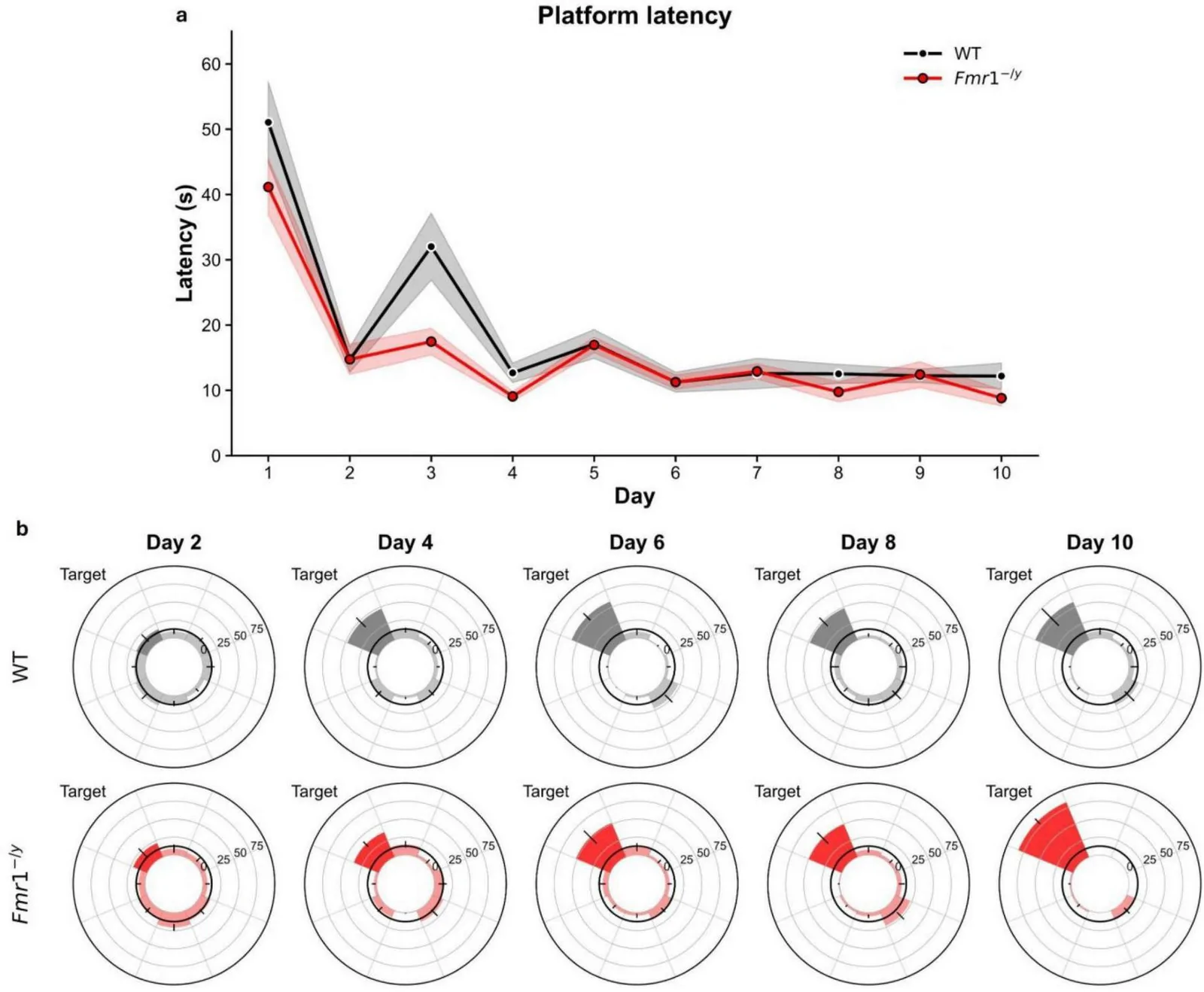
Daily platform latency and alternate day probe sessions in Fmr1^–/y^ and WT rats. **(a)** Both *Fmr1*^–/y^ and WT rats showed decreased latencies to find the hidden platform across testing days. **(b)** Both *Fmr1*^–/y^ and WT rats showed increasing bias toward the correct octant during platform removal probe sessions conducted every other day. Data points are means and shading indicates standard deviations. Interior lines indicate percentages of time in octants. The interior black circle indicates chance level.

In the platform removal probe trials run on every other day, both WT and *Fmr1*^–/*y*^ rats showed an increase in the target octant occupancy across days, with their target octant occupancy increasing significantly from day 2 to 10 [*F*(4,44) = 6.82, *p* < 0.001]. There was no significant difference between the two groups [*F*(1,11) = 0.54, *p* = 0.48]. Both WT and *Fmr1*^–/*y*^ rats showed a preference for the target octant by day 4 [WT: *t*(7) = 3.81, *p* = 0.003, *Fmr1*^–/*y*^: *t*(6) = 4.93, *p* = 0.001; see Figure 3b].

In the landmark removal probe session, three of the four landmarks were removed and only the lantern was kept. There was no significant difference between WT and *Fmr1*^–/*y*^ rats in terms of target octant occupancy [*U*(7, 8) = 28, *p* = 1], though WT rat occupancy failed to reach significance [*t*(6) = 1.87, *p = 0.055*] while occupancy for *Fmr1*^–/*y*^ rats was significant [*t*(7) = 2.6, *p* = 0.018] (Figure 4; data for one WT rat for this probe was lost due to technical issues during the trial). To explore this difference, a second landmark removal probe was conducted with only the white board remaining. Both WT and *Fmr1*^–/*y*^ rats showed a preference for the target octant [WT: *t*(6) = 4.89, *p* = 0.001; *Fmr1*^–/*y*^: *t*(7) = 3.71, *p* = 0.004] and there was no difference between the two groups [*U*(7, 8) = 35, *p* = 0.46]. The second single cue probe was conducted 4 weeks after the first, with 1 day of normal training with all landmarks present preceding the probe on the previous day.

**FIGURE 4:**
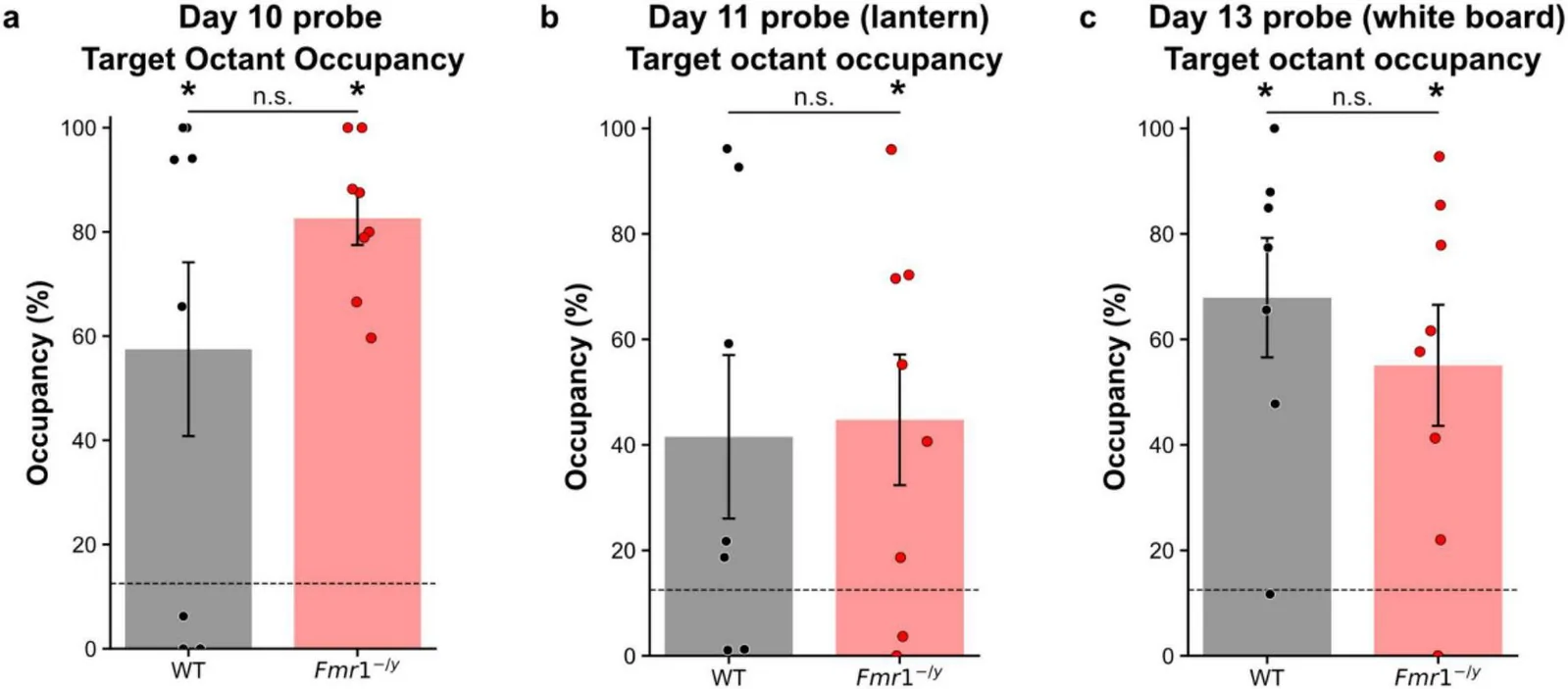
Octant occupancy at the end of training and on landmark removal probe sessions for *Fmr1*^–/y^ and WT rats. **(a)** Both *Fmr1*^–/y^ rats and WT rats showed a bias toward the correct octant in the probe session at the end of training, and in probe sessions **(b,c)** with three of the four landmarks removed (center plot: lantern landmark; right plot: white board landmark). Data are means and standard error; data points are individual animals. Statistical significance (*p* < 0.05) is indicated with a * and the dashed line indicates chance level.

### 3.3 *Syngap1*^+/–^ rats show robust learning in a directional water maze task

The directional water maze task was conducted on two cohorts of male *Syngap1*^+/–^ and WT rats; the first cohort (WT = 5, *Syngap1*^+/–^ = 5) were the animals previously used in a pilot version of the water maze (data not shown). The second cohort (WT = 6, *Syngap1*^+/–^ = 3) was trained from the start in the directional version of the water maze where they received visible platform training trials on the first 2 days. Across 10 days of training, both *Syngap1*^+/–^ (*n* = 8) and WT animals (*n* = 11) showed a significant decrease in time to find the submerged platform [main effect of day, F(9,153) = 13.7, *p* < 0.001; Figure 5]. There was also an overall significant genotype effect with *Syngap1*^+/–^ rats taking longer to find the platform [*F*(1,17) = 9.57, *p* = 0.007], but no interaction between genotype and day [*F(*9,153) = 0.87, *p* = 0.55].

**FIGURE 5:**
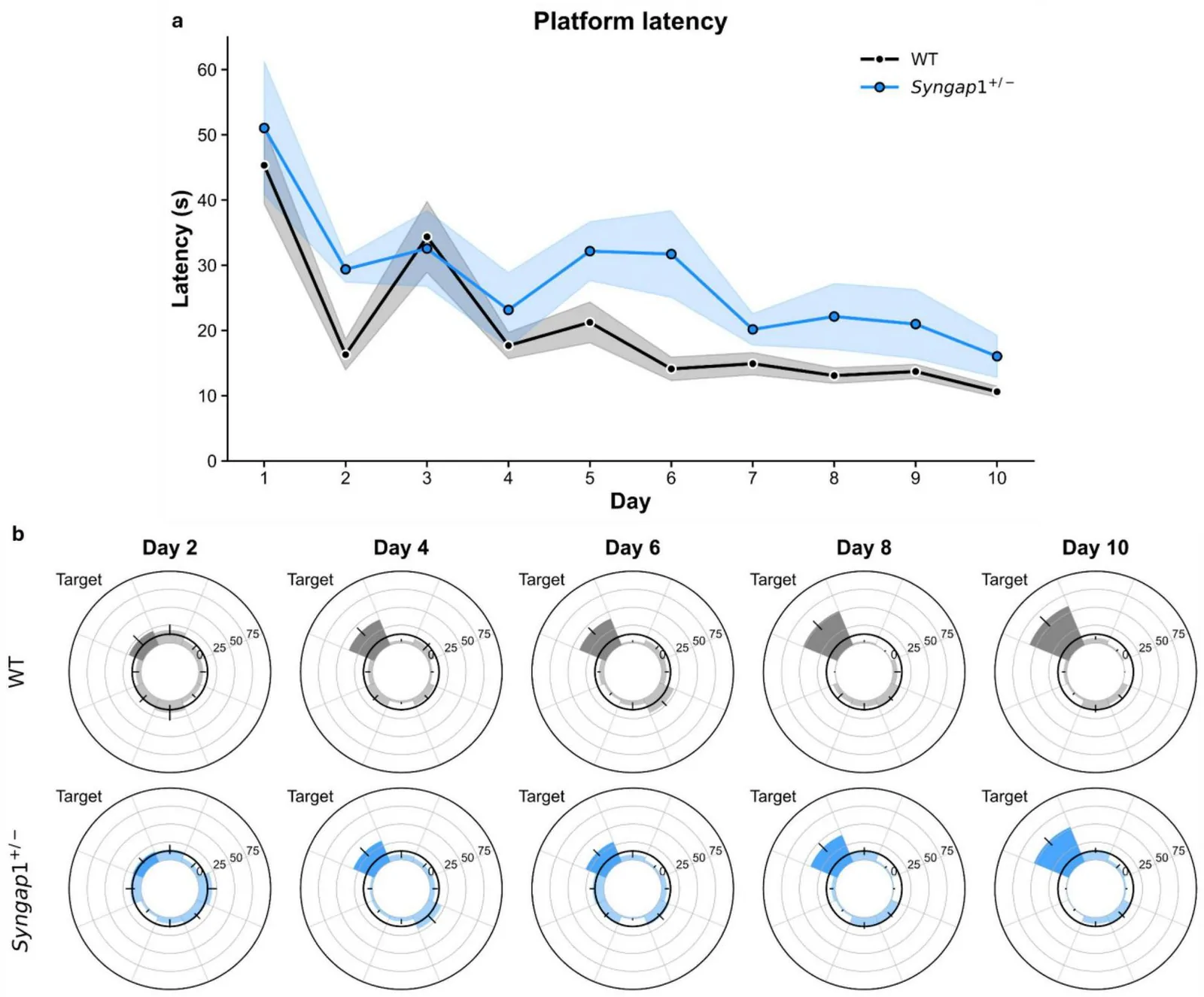
Platform latency and daily probe performance of *Syngap1*^+/–^ and WT rats. **(a)** S*yngap1^+/–^* rats differed from WT rats in their latencies to find the submerged platform in training (top graph). Lines are means and shading indicates standard error. **(b)** The two groups did not differ in their preference for the platform octant across the probe sessions occurring every other day across training. Data are means and standard errors, and darker shading in each plot indicates target octant. Interior lines indicate percentages of time in octants. The interior black circle indicates chance level.

In the probe sessions (the first trial of every second day of training), there was no significant difference between *Syngap1*^+/–^ rats and their WT littermates [*F*(1,8) = 0.66, *p* = 0.44]. Overall, both groups showed evidence of directional learning with target octant occupancy on the probe trials increasing significantly across days [*F*(4,32) = 5.94, *p* = 0.001]. There was no significant interaction between genotype and day, [*F*(4,32) = 0.34, *p* = 0.85]. Both groups of rats showed a preference for the target octant by day 4 [*T*-test against chance level performance: WT: *t*(10) = 3.29, *p* = 0.004, *Syngap1*^+/–^: *t*(7) = 2.38, *p* = 0.024; see Figure 5b].

To test whether the rats required all landmarks to be present, upon completion of training a probe session was run in which three of the four visual landmarks were removed and the submerged platform was unavailable. Different cues were removed for the two cohorts, and in both cohorts WT rats showed a significant preference for the target octant [Cohort 1: *t*(4) = 2.22, *p* = 0.045; Cohort 2: *t*(5) = 3.1, *p* = 0.014] while the *Syngap1*^+/–^ rats did not [Cohort 1: *t*(4) = 1.75, *p* = 0.077; Cohort 2: *t*(2) = 2.17, *p* = 0.081], but no significant difference was observed between the two groups [Cohort 1: *U*(5, 5) = 16, *p* = 0.55; Cohort 2: *U*(6, 3) = 11, *p* = 0.69].

### 3.4 *Grin2b*^+/–^ rats show robust learning in a directional water maze task

As with the *Syngap1*^+/–^ and the *Fmr1*^+/*y*^ rats, *Grin2b^+/–^* rats showed rapid decreases in their latencies to find the hidden platform that mirrored those of WT rats. There was no overall difference between the groups [*F*(1,10) = 0.021, *p* = 0.89] and no interaction between group and training day [*F*(9,90) = 1.44, *p* = 0.18]. There was an overall effect of training day [*F*(9,90) = 14.39, *p* < 0.001].

As in the other two models tested, both the WT and *Grin2b^+/–^* rats showed increased target octant occupancy across the platform-removal probe sessions, increasing from day 2 to 10 [*F*(4,40) = 5.33, *p* = 0.002]. Again, there was no significant main effect of genotype [*F*(1,10) = 0.5, *p* = 0.49] or interaction between genotypes and probe session [*F*(4, 40) = 0.993, *p* = 0.42]. Interestingly, both groups of rats showed a preference for the target octant by day 2 [WT: *t*(5) = 2.28, *p* = 0.036, *Grin2b^+/–^*: *t*(5) = 3.36, *p* = 0.01; see Figure 7], as opposed to day 4 seen with the *Syngap1*^+/–^ and the *Fmr1*^+/*y*^ rat models.

For the visual landmark removal probes, three probe sessions were run, each with a different remaining landmark (Figure 8). In the first probe, the sombrero hat was kept and neither group of rats showed a preference for the target octant [WT: *t*(5) = −2.72, *p* = 0.99, *Grin2b^+/–^*: *t*(5) = 0.93, *p* = 0.39] with no significant difference between the two groups [*U*(6, 6) = 16, *p* = 0.78]. It is notable that in this probe session, no WT animals showed an above chance preference for the correct octant, while two *Grin2b^+/–^* rats spend > 80% of their octant-dwelling times in the correct octant. This result suggests that for a subset of the *Grin2b^+/–^* rats, this specific landmark was sufficient to guide directional preference.

The second single cue probe trial (cue retained: white board) was conducted a week after the first, with a normal day of training on the preceding day with all four landmarks present. The WT had a significant preference for the target octant [t(5) = 3.51, *p* = 0.009], while preference in the *Grin2b*^+/–^ rats approached, but did not reach significance [t(5) = 1.87, *p* = 0.061], and there was no significant difference between the two groups [U(6, 6) = 21, *p* = 0.68]. The third single cue probe trial (cue retained: lantern) was conducted a week after the second, with a normal day of training on the preceding day with all four landmarks present. Both the groups of rats showed a preference for the target octant [WT: t(5) = 4.45, *p* = 0.003; *Grin2b*^+/–^: t(5) = 2.81, *p* = 0.019;] for this probe with no significant difference between the two groups [U(6, 6) = 22.5, *p* = 0.52]. As discussed below, these results may be indicative of the use of associations between the platform location and individual visual landmarks (as opposed to the complete constellation of landmarks) for directional learning in this task.

## 4 Discussion

The current results indicate that rats can readily learn to associate a submerged platform’s direction in a version of the water maze with controlled visual landmarks. Although we observed differences between WT animals and *Syngap1*^+/–^ (slower) and *Fmr1*^+/*y*^ rats (faster) in their latencies to find the platform across training, these differences were not observed in the arguably more robust measure of octant bias during platform removal probe sessions. No differences in either latency or octant bias were observed between *Grin2b^+/–^* rats and their WT littermates. The landmark removal probe sessions suggest that individual landmarks, with one exception, were sufficient to control directional behavior. These results are considered in detail below.

### 4.1 Intact spatial learning in that *Fmr1*^–/*y*^, *Syngap1*^+/–^, and *Grin2b*^+/–^ rats

The current results indicate that all three models of ASD/ID learn the association between the visual landmarks and the submerged platform direction readily. As noted in the Introduction, studies with *Fmr1*^–/*y*^ mice have yielded mixed results in terms of spatial impairments. The limited results in rats appear, however, to be more consistent: the current study, and the previous work by Till et al., 2015 and Tian et al., 2017 together show that *Fmr1*^–/*y*^ rats are unimpaired in learning a submerged platform location or direction when assessed in platform removal probe sessions. The results are also unsurprising given the over-reliance of the head direction system on visual landmarks that we have recently observed in *Fmr1*^–/*y*^ rats (Duszkiewicz et al., 2025), though place cell spatial information is decreased across days in these animals (Asiminas et al., 2022).

A similar pattern of results is seen across studies with *Syngap1*^+/–^ animals. While spatial impairments have been observed in *Syngap1*^+/–^ mice, the current results agree with those of Katsanevaki et al. (2024) in showing the *Syngap1*^+/–^ rats readily learn the location of a submerged platform in a water maze as measured by probe trial performance. The impairment in visual discrimination abilities subsequently reported by Katsanevaki et al. (2025) was not evident in the learning of the visual landmarks in the current study, though quite salient landmarks were used by design. The lack of spatial learning impairment in *Syngap1*^+/–^ rats is intriguing, given their reduced capacity for LTP (Katsanevaki et al., 2024).

In the sole previous paper with GluN2B-trp373 variant rat model (Wang et al., 2022), an impairment in spatial learning on the water maze was claimed with the GluN2B mutation rats compared to WT rats. However, this conclusion was not based on the common measure of spatial memory in the platform removal probe, dwell time in the quadrant where the platform had been present in training, but instead on platform crossings. For this measure of spatial learning, the difference was modest as both WT animals and GluN2B animals were within a similar range (2–3 crossings). The current results, with repeated probe sessions, provide more robust evidence of intact spatial learning and memory in *Grin2b^+/–^* rats, comparable to that of WT littermates.

Despite the diversity of systems affected by these gene alterations, which range from RNA binding to receptor subunit expression, the intact learning in these animal models stands in contrast to significant learning impairments observed in humans with the same alterations. This contrast may suggest that more (1) basic, associational processes such as the learning of landmarks are spared in these genetic conditions, and (2) higher order, sensory conflict tasks, may be required to reveal deficits in these animal models. For example, when only a single source of information (e.g., a visual landmark) is sufficient to guide performance, inflexibility in integrating other types of sensory information may not be apparent.

### 4.2 Annular strategy in an open water maze

The modified water maze used in the current study was developed following pilot observations that rats in an open water maze could employ an annular swimming strategy to find the submerged platform with controlled landmarks that were moved each day (see inset in Figure 2C). Such a strategy would not require reliance on extra-maze landmarks to find the platform, but could rather reflect learning the distance of the platform from the maze walls. This strategy has been referred to as circling (Graziano et al., 2003) or “chaining” (Wolfer and Lipp, 2000), and such a ring-search strategy is more persistent during water maze acquisition in older rats (Srivathsa et al., 2026). An intriguing possibility for why rats may have employed such a strategy in our pilot work is that when access to extramaze cues is limited, either with diminished sensory capacity in older animals or with the provision of a limited number of landmarks available (as in the current experiment), learning to use extramaze landmarks to find the submerged platform is more challenging and animals employ less “spatial,” but partially effective strategy such as an annular search.

### 4.3 Stimulus control in the water maze

As a secondary finding, one of the goals of the current work was to demonstrate explicit stimulus control of spatial learning and memory via visual landmarks. In the water maze as it is traditionally run, the spatial association is assumed to be between the position of the submerged platform and distal visual landmarks in the laboratory room. In such a situation, it is not possible to specify which landmark or constellation of landmarks the rat might be using to triangulate the submerged platform’s location. The final version of the water maze used in the current experiment overcame this limitation by (1) limiting the number of polarizing visual landmarks to four, within a curtained enclosure, and (2) shifting the landmarks and the submerged platform in register daily. The latter procedure dissociated the platform location from uncontrolled landmarks in the environment and rendered the controlled landmarks as the most reliable external cues to the submerged platform’s direction. To our knowledge, only Rodrigo et al. (1997) have previously used such an approach. Rats readily learned the platform location - in as few as 2–4 days of training - under these circumstances, as evidenced by the platform removal probe sessions. This result is consistent with previous findings that rats and mice can learn the platform location with a controlled set of extramaze landmarks (e.g., Fenton et al., 1994a,^1995^; Rodrigo et al., 1997; Nakazawa et al., 2002).

A central assumption of spatial learning on the water maze is that rats use a constellation of landmarks to triangulate the position of the submerged platform. This assumption is not regularly tested (with the exceptions Fenton et al., 1994a; Rodrigo et al., 1997; Nakazawa et al., 2002), and instead is assumed by the availability of multiple visual landmarks in the typical experimental room. The specification of landmarks in the current experiment allowed assessment of the constellation vs. individual cue strategy for identifying platform direction. If a constellation of landmarks is essential, removal of a subset of these should impair performance. In contrast, if individual landmarks are used, one would predict that the animals using the remaining landmark (in the probes where the remaining landmarks are removed) would perform well, while those using a different landmark would perform at chance levels.

The landmark removal probe sessions - in which three of the four trained landmarks were removed - revealed performance that is consistent with the use of individual landmarks to identify the correct platform octant. We observed that performance overall was above chance levels for each group for almost every individual landmark probe session, with the exception of the *Syngap1*^+/–^ rats (which approached, but did not reach statistical significance). It is possible that this lack of octant preference in *Syngap1*^+/–^ rats is due to an impairment in pattern completion when only a subset of trained landmarks was available. However, in most single landmark sessions, a subset of both WT and *Syngap1*^+/–^, *Fmr1*^+/*y*^, and *Grin2b^+/–^* rats were at or below chance levels of occupancy for the target octant. A clear example of this result is seen in Figure 6, where a subset of *Syngap1*^+/–^ rats showed no preference for the correct octant when there was only one landmark present, while two others within this group showed > 80% occupancy in this same session. What this pattern suggests is that different animals were relying on different individual landmarks to identify the correct octant, as use of a constellation of landmarks would have instead resulted in impaired performance for all single-landmark probe sessions. An acknowledged weakness of the current study, however, was that not all groups of rats were tested with all individual landmarks.

**FIGURE 6:**
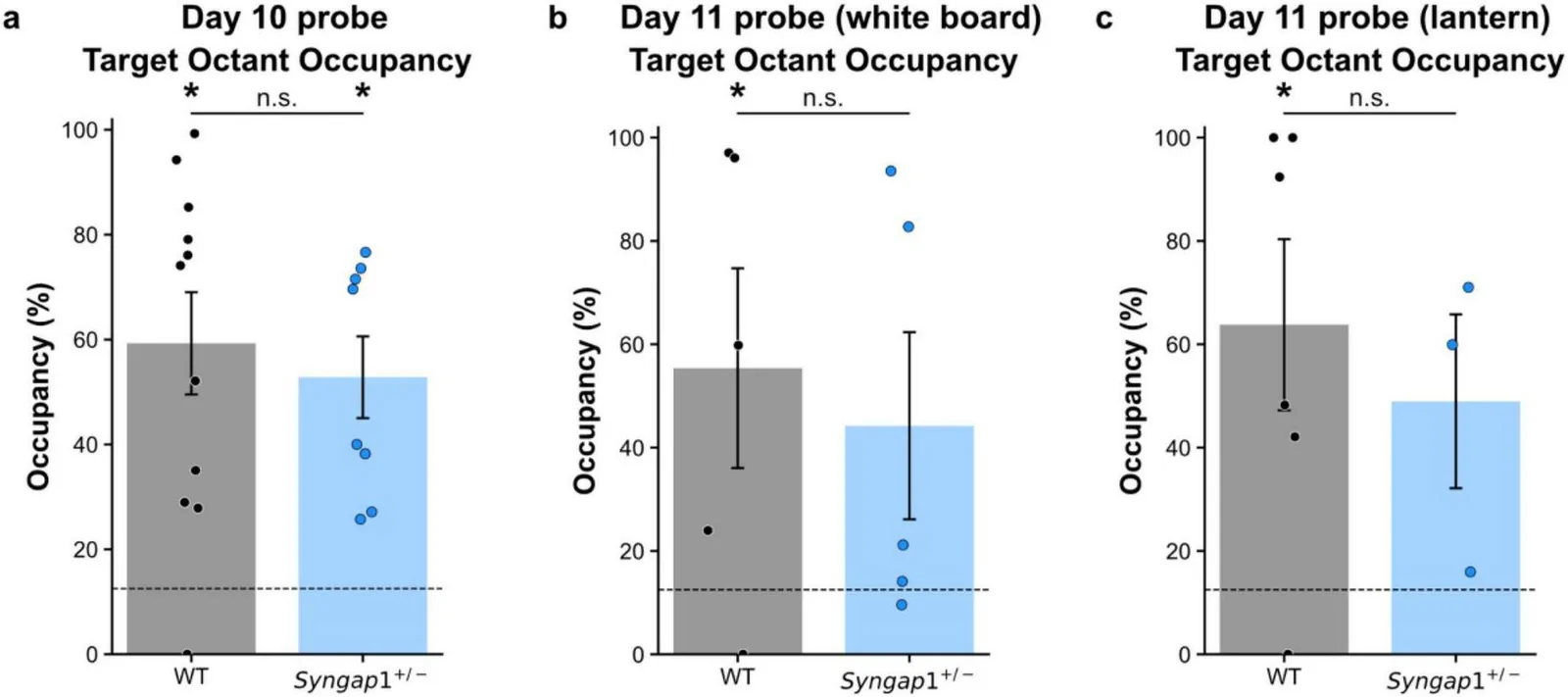
Octant occupancy at the end of training (Day 10) and on landmark removal probe sessions for *Syngap1*^+/–^ and WT rats. **(a)** Both *Syngap1*^+/–^ and WT rats showed a substantial preference for the correct octant in the final (all landmarks present) probe session on day 10, while only WT rats showed a significant preference for the correct octant in probe sessions **(b,c)** in which three of the four landmarks were removed. Statistical significance (*p* < 0.05) is indicated with a * and the dashed line indicates chance level.

**FIGURE 7:**
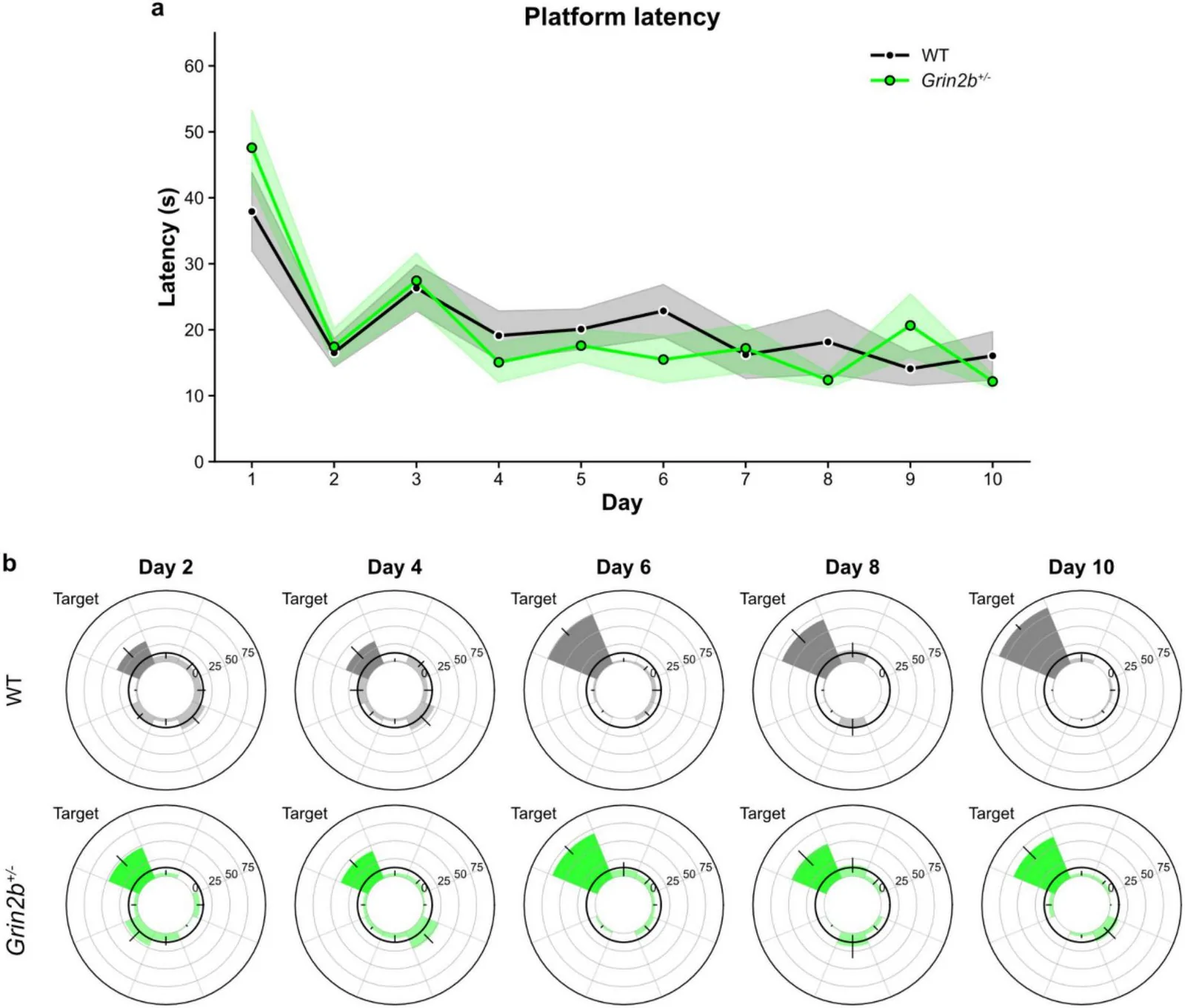
Daily platform latency and alternate day probe sessions in *Grin2b^+/–^* and WT rats. **(a)** Both *Grin2b^+/–^* and WT rats showed a decrease in latencies to find the submerged platform across training trials. **(b)** Both *Grin2b^+/–^* and WT rats showed an increase in bias to the correct octant in the probe sessions run every other day in training. Data points are group means and shading indicates standard errors. Interior lines indicate percentages of time in octants. The interior black circle indicates chance level.

**FIGURE 8:**
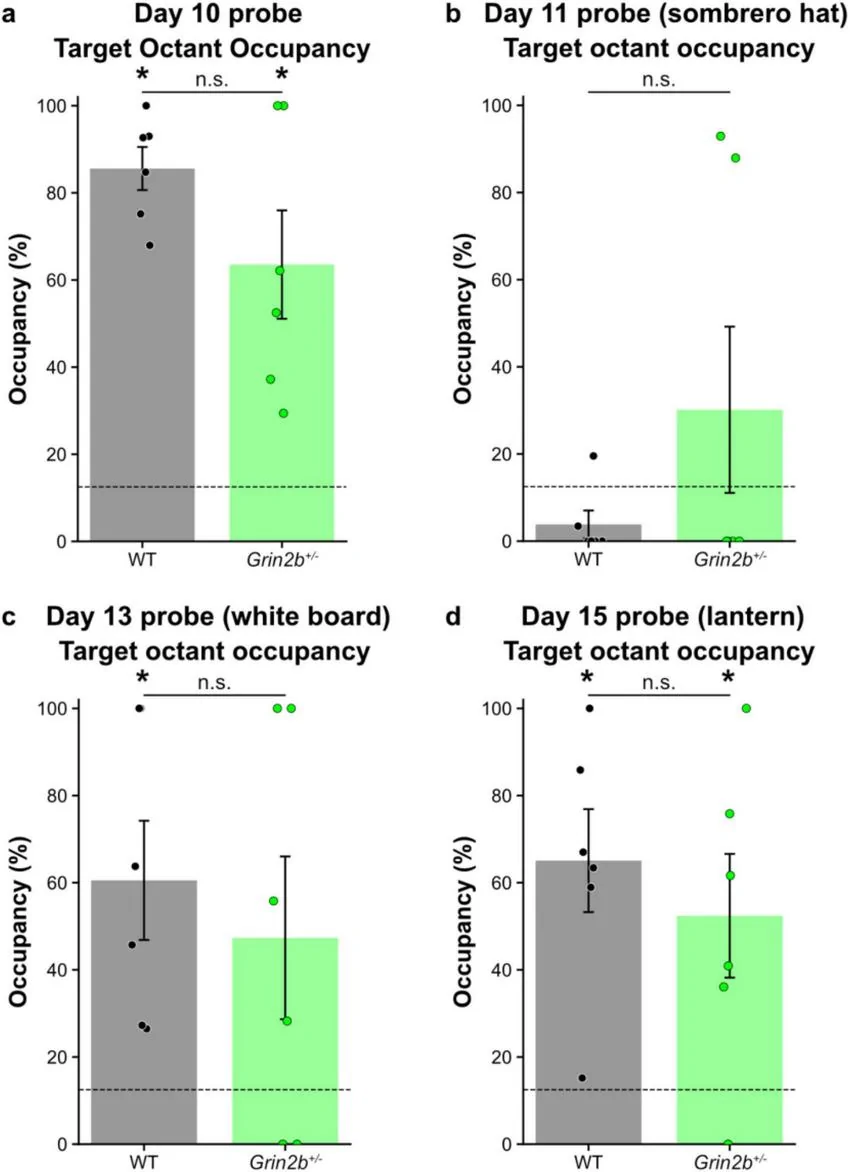
Octant occupancy at the end of training and on landmark removal probe sessions for *Grin2b^+/–^* and wild type rats. **(a)** G*rin2b^+/–^* and WT rats showed a high preference for the correct octant in the final standard probe session (with all landmarks available). Statistical significance (*p* < 0.05) is indicated with a *. **(b)** Target octant occupancy for probe trials with three out of four landmarks removed (landmark remaining: sombrero hat). Neither the WT or the *Grin2b^+/–^*^–^ rats showed a significant preference for the target octant (occupancy was not significantly different from chance) for this condition, with no significant difference between the two groups. **(c)** Target octant occupancy for probe trials with three out of four landmarks removed (landmark remaining: white board). WT occupancy was significantly above chance level, whereas *Grin2b^+/–^* rats approached significance (*p* = 0.06), with no significant difference between the two groups. **(d**) Target octant occupancy for probe trials with three out of four landmarks removed (landmark remaining: lantern). Both the WT and *Grin2b^+/–^* rats showed target octant occupancy significantly greater than chance, with no significant difference between the two. The dashed line indicates chance level.

Nonetheless, these findings are consistent with earlier study by Fenton et al. (1994a) who showed that any two of four controlled landmarks were sufficient to maintain performance on a water maze. In a subsequent study, Fenton et al. (1995) found impaired performance when only one familiar landmark was available (but paired with a second, unfamiliar landmark; see also Rodrigo et al., 1997; Prados and Trobalon, 1998). Two differences in these previous studies and the current study may be relevant, however. First, in these studies, rats were trained in an open maze, whereas in the current study animals were constrained to alleyway “choices.’ Thus, it might be that an elemental or “snapshot” learning is sufficient when choices are constrained, as in the present study. Arguing against this interpretation, however, is the finding that rats show impaired spatial performance on a radial maze, where choices are likewise constrained, when only one external landmark is available (Pico et al., 1985; but see Dudchenko and Taube, 1997). Secondly, though the animals in the Fenton study took longer to find the hidden platform when using only one familiar cue (+ an unfamiliar cue) was available vs. pairs of familiar cues, these latencies were considerably less than those observed when the animals were retrained on a new platform location. Similarly, Prados and Trobalon (1998) reported a trend for rats to spend more time and cover more distance in the correct quadrant of the water maze when only one of four trained landmarks was available, though this preference did not reach statistical significance. Thus, in both studies, performance was impaired when only one familiar cue was available, but some capacity to locate the submerged platform may still have been present. Finally, in mice, intact spatial memory has been shown with a single landmark following training with four landmarks (Nakazawa et al., 2002).

## 5 Summary

The current study shows that in three models of neurodevelopmental disorders - *Fmr1*^–/*y*^, *Syngap1*^+/–^, and *Grin2b*^+/–^ rats - landmark learning is unimpaired compared to that of WT animals. Second, we demonstrated that rats can learn to locate a submerged platform direction based on controlled visual landmarks. Finally, our results imply that animals may rely on directional associations with individual landmarks to perform this task, despite training with a constellation of landmarks. This last finding may suggest that a form of pattern completion when only a subset of learned landmarks are available may be sufficient for above-chance performance, akin to the demonstration that the place fields of hippocampal place cells remain intact when only a subset of trained landmarks are available (Nakazawa et al., 2002).

## Author’s note

Anuj Vadher, Adrian Duszkiewicz and Paul Dudchenko are at the Division of Psychology, University of Stirling, Stirling, U.K. Adrian Duszkiewicz is also at the Division of Neuroscience, University of Manchester, Manchester, U.K. Emma Wood is at the Institute for Neuroscience and Cardiovascular Research and Simons Initiative for the Developing Brain, University of Edinburgh, Edinburgh, U.K. The authors would like to acknowledge the assistance of Ms Manaswini Agnihotri in testing a subset of the animals in this work, and the Bioresearch and Veterinary Services staff, University of Edinburgh for their care and support for the animals used in the experiments.

A preliminary version of these results was presented at the Society for Neuroscience annual meeting in 2025 (Abstract number LBP033.07/LBP110).

## Data Availability

The raw data supporting the conclusions of this article will be made available by the authors, without undue reservation.
